# Effect of Low-Level Cyclic Loading on Bond Behavior of a Steel Bar in Concrete with Pre-Existing Damage

**DOI:** 10.3390/ma14227080

**Published:** 2021-11-22

**Authors:** Chongku Yi, Jeeho Lee, Kee-Jeung Hong

**Affiliations:** 1 School of Civil, Environmental & Architectural Engineering, Korea University, Seoul 02841, Korea; chongku@korea.ac.kr; 2 Department of Civil & Environmental Engineering, Dongguk University, Seoul 04620, Korea; 3 School of Civil & Environmental Engineering, Kookmin University, Seoul 02707, Korea

**Keywords:** bond-slip, concrete, steel rebar, cyclic loading, numerical model

## Abstract

Understanding the bond behavior of steel rebar in concrete is important in order to determine the performance of a reinforced concrete structure. Although numerous studies have been carried out by many researchers to develop a robust model for numerical analysis, no consensus has been reached as the bond behavior depends on hysteresis. In this study, the bond behavior of a steel bar in concrete with pre-existing damage is investigated under low-level cyclic loading. Based on the experimental bond stress and slip curve, a numerical model for finite element analysis to simulate the effect of low-level cyclic loading is proposed. The results from the numerical analysis show good agreement with the experimental data, including accumulated damage on stiffness and strength throughout entire load cycles.

## 1. Introduction

Bonding behavior of steel bars in concrete has been studied by several researchers in recent decades. In 1960s and 1970s, most of experimental studies focused on the bond behavior under monotonic loading with different factors as variables: rebar deformation [1], boundary condition [2], deformation dimensions [3], and cover thickness and concrete strength [4]. Analytical models needed to use refined numerical method to determine reinforced concrete behavior were also reported [4,5]. The effect of hysteresis on the bond behavior of a steel bar in concrete was first reported in the early 1970s [6], and a number of studies reported in the 1980s [7,8,9] laid groundwork for a numerical model. However, there has been no single model that reflects all factors that affect the bond stress–slip behavior of steel rebar in concrete due to difficulties in measuring bond stress and bond slip, and unpredictable hysteresis and boundary conditions.

**Figure 1:**
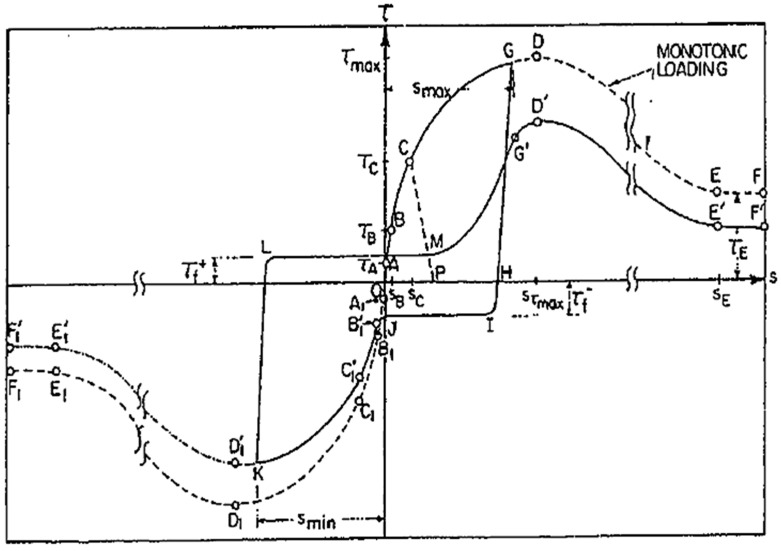
Typical relationship between bond stress ( τ ) and slip ( s ) for monotonic and cyclic loading [8].

Under cyclic loading, the degradation of bonding behavior is more severe than that under monotonic cyclic loading. The degradation becomes more significant with larger slip value at which reversal of loading occurs. Ismail and Jirsa [7] found that application of load cycles with constant peak stress can yield gradual deterioration in bonding even with a small number of repetitions. Eligehausen et al. [8] carried out an experimental study to investigate the bond stress–slip relationship under cyclic loading with an extensive list of variables. They showed that the envelope of bond stress–slip hysteresis loops for repeated loading lies below the curve from monotonic loading, as shown in Figure 1. Gan listed the most important parameters under cyclic loading as follows: strain (or stress) range, type and rate of loading (strain rate), and the value of maximum imposed bond stress [10]. Based on the test results, the bond stress–slip models were proposed according to two basic approaches: (1) microscopic approach reflecting the local stress state between concrete and rebars [11,12], and (2) a macroscopic approach simulating the global behavior of a member [13,14,15].

Due to low-level cyclic loading, such as the moving service loads or small earthquakes, a cumulative damage to the bond between steel rebar and concrete can occur. Since this accumulated damage can compromise the seismic performance of reinforced concrete structures, its effect on bond behavior of steel bar in concrete needs to be understood. In the present study, this effect is investigated by constant amplitude cyclic bond tests under displacement control with a fixed number of loading cycles. In addition, a numerical model for finite element analysis to simulate the effect of low-level cyclic loading is derived based on the experimental result.

## 2. Experiment

### 2.1. Materials

**Figure 2:**
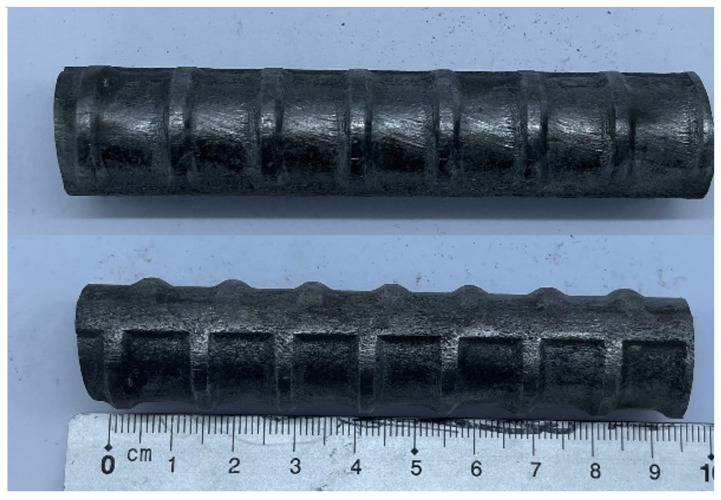
Image of SD400 D19 steel rebar used in this study.

The steel rebar used in this study was manufactured by a local company (Dongkuk Steel Mill Co., Ltd., Seoul, Korea) according to specifications provided in Korean Industrial Standard KS D 3504 [16]. Figure 2 shows a sample of the rebar. The nominal diameter of the rebar was 19 mm and graded as SD400 (minimum yield strength of 400 MPa, minimum tensile strength of 560 MPa). The chemical composition of steel bar was tested according to KS D1801 [17]. Table 1 shows the analysis results, which confirms that the chemical composition of the steel bar satisfies the specifications per KS D 3504. Concrete was prepared by a local ready mixed concrete manufacturer. The strength of concrete was designed as 27 MPa, the slump was 120 mm, the water–cement ratio was 48.2%, the maximum size of aggregate was 25 mm, and the air volume was 4.5%. The concrete composition is shown as Table 2.

### 2.2. Specimen Preparation

**Figure 3:**
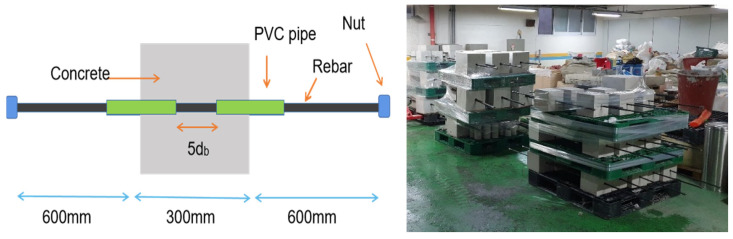
Dimensions of the Specimen (**left**), image of specimens (**right**).

As shown in Figure 3, test specimens were designed as a cube having sides dimension equal to 15 times the diameter of the bar (15d_b_, 300 mm) to avoid splitting failure with embedment length of 5d_b_, based on the study by Casanova et al. [18].

Steel bars were cut to equal length of 1800 mm. The length of the steel bar was determined in order to acquire room for LVDT (Linear Variable Differential Transformer) installation and screw thread for mechanical coupler, which was adapted to anchor the rebar during the test. Threads were made at both ends of the steel bar over the length of 60 mm according to the specification set by the coupler manufacturer (Jungwoo B&C Co., Ltd., Paju, Korea).

The embedded length (5d_b_) was secured in the middle of the reinforcement guided by two PVC pipes of which openings were later sealed with silicone to prevent intrusion of concrete into the pipe during concrete placement, as shown in Figure 3. Other parts of reinforcement were sprayed with WD-40 (Water Displacement 40th formula by WD-40 Company, San Diego, CA, USA) to prevent corrosion. After the concrete placement, all specimens were cured in moist condition for up to 28 days.

### 2.3. Cyclic Load Test Method

**Figure 4:**
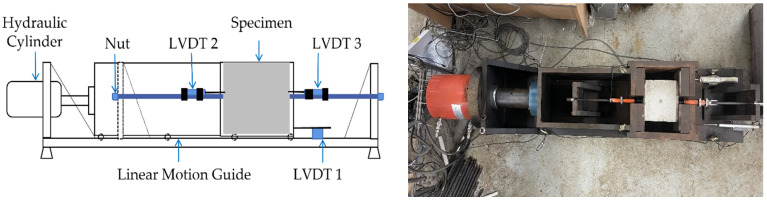
The schematic drawing of the test set-up (**left**) and actual image of the set-up (**right**).

The testing machine shown in Figure 4 was used for cyclic load test. The loading device was designed to exert the load on the specimen in two different modes: displacement control or load control. The double acting hydraulic cylinder with 200-tonf capacity at pressure of 700 bar was manufactured by a local company (TDC-20015 by Daejin Hydraulic Machinery Co., Ltd., Busan, Korea). A low-profile load cell with a 20-tonf compression and tension measurement range, manufactured by a local company (Bongshin Loadcell Co., Ltd., Osan, Korea), was used to measure applied load. Two linear displacement transducers (CDP-50M by Tokyo Measuring Instruments Lab Co., Ltd., Tokyo, Japan) were used to measure local slip as shown as LVDT2 and LVDT3 in Figure 4. An additional linear displacement transducer, LVDT1, was installed on the base plate to monitor the global displacement of the specimen holder. All measurements were recorded using a data logger (UCAM-60B by Kyowa Electronic Instruments Co., Ltd., Tokyo, Japan) at one second intervals.

After placing a specimen inside the specimen holder by hand, each specimen was positioned and secured as follows: (a) thin steel sheets (0.5 mm, 1 mm, 2 mm thickness) were used as shim plate to secure the specimen in the middle of the holder as needed; (b) the position of the specimen was then adjusted using the hydraulic cylinder so that the specimen sits at the center between the reaction supports; (c) the initial readings from the LVDT and the load cell were recorded, and the steel bar was fastened with a set of semi-spherical washer and a rebar coupler at each end. The load was monitored during the fastening so that it remained consistent with the initial load reading. 

A monotonic pullout test was performed first by applying displacement load at the rate of 0.03 mm/s until the average local slip value reached 20 mm in order to determine the low-level cyclic loading parameters. From the monotonic pullout test, the average local slip corresponding to the maximum bond stress, S_m_, was found to be 1.67 mm. Based on the S_m_ obtained from the monotonic test, the cyclic load level for this study was determined as (±)0.5 mm, which equals to (±)0.3 × S_m_.

**Figure 5:**
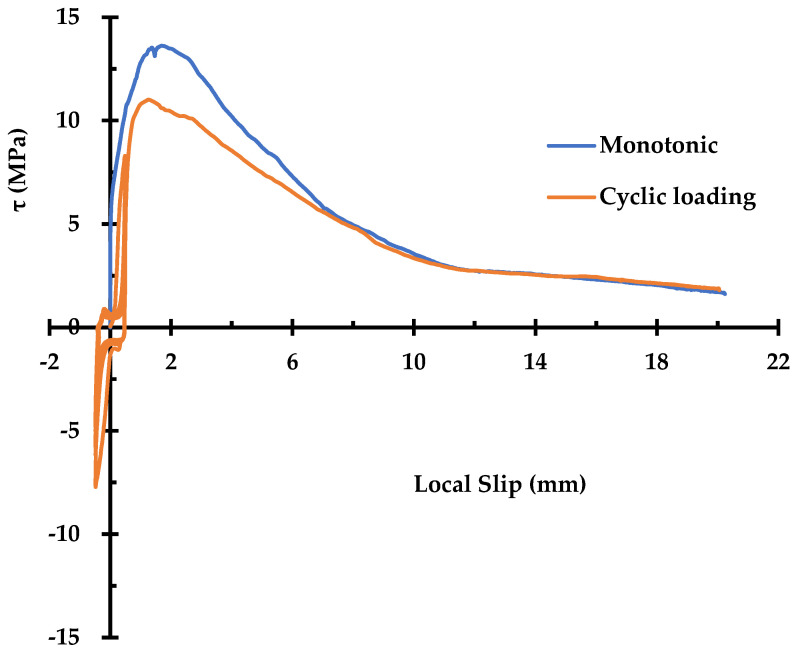
Pullout behavior of the steel rebar with and without the cyclic loading.

Before the commencement of the cyclic load test with the (±)0.5 mm load level, in order to emulate the pre-existing damage in a reinforced concrete structure, the specimen was subjected to three cycles of load in the range of (−)0.5~(+)0.5 tonf, which showed no measurable local displacement, followed by four cycles in the range of (±)1 tonf which resulted in a discernable local displacement. Afterward, the (±)0.5 mm load was applied 10 times under the displacement control, at a rate of 0.03 mm/s, to record bond-slip behavior. Upon completion of 10 load cycles, the pullout behavior of the steel bar was recorded up to the local slip value of 20 mm in the same way as for the monotonic pullout test specimen as mentioned above. The pullout result is shown in Figure 5.

### 2.4. Experimental Result and Discussion 

**Figure 6:**
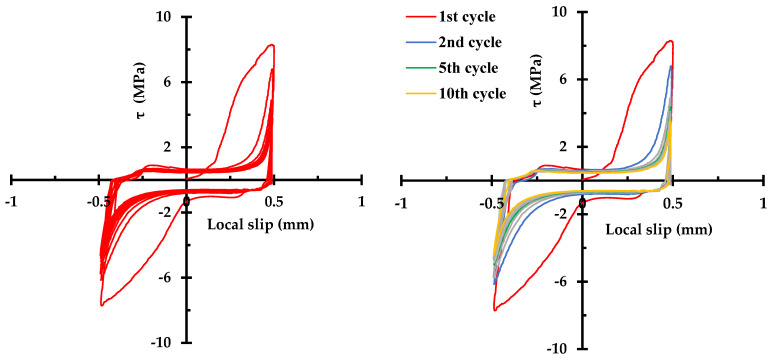
Bond stress–slip curve: all 10 cycles are shown (**left**), selected cycles shown to accentuate the effect of the cyclic load on the bond stress–slip behavior (**right**).

Figure 6 shows that the ascending branch of the first cycle begins with a shallow slope and the slope sharply increases with the inflection point at the bond stress value of 4.5 MPa. The ascending branch softens gradually and reaches the maximum bond stress of 8.3 MPa at the slip of 0.5 mm. Upon unloading, the load drops rapidly with a little change in slip with the slope of 200 MPa/mm. As the displacement load increases in reverse direction, bond stress increases very slowly up to (−)1.03 MPa, at which point a plateau is observed until the slip value reached zero. It is noted that the plateau is consistent with a frictional resistance; with increase in the displacement, bond stress increases with the inflection point at about (−)3 MPa. At the slip value of (−)0.5 mm, the bond stress reached (−)7.7 MPa, which is 6% less than the maximum bond stress at (+)0.5 mm. As the displacement direction is reversed, the load drops to zero with 0.1 mm change in slip and exhibits a new plateau at bond stress value of (+)0.8 MPa until the slip reaches zero. The reduction in frictional resistance and the slope of the descending branch of the curve in the negative slip range can be attributed to the cumulated damage to the bond between the concrete and steel.

**Figure 7:**
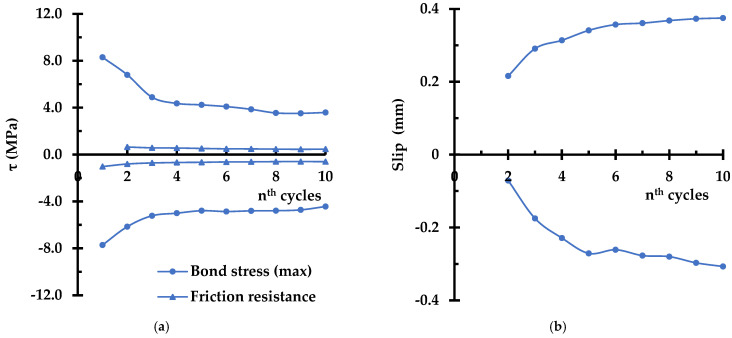
(**a**) Maximum bond stress in nth load cycle, (**b**) slip corresponding to the end of friction plateau for nth load cycle.

The general shape of the descending branch is retained for the subsequent load cycles (second to tenth), though it is noted that there is no inflection point in the ascending branch. The absence of the inflection point in the ascending branch suggests that the slip-resistant mechanism is consolidated to a single mode after the first load cycle. As can be seen in Figure 7a, the maximum bond stress (τ_m_) and the bond stress corresponding to the frictional resistance (τ_f_) show significant degradation (41% and 30%, respectively) in the first three cycles, and approach asymptotic values with final degradation of 57% and 41% at the tenth cycle, with respect to those of the first cycle. Furthermore, it is noted that frictional resistance is always lower in positive direction than in negative direction by 21% on average. Figure 7b also shows asymmetry of the frictional plateau. The directionality of the bond–slip behavior seems to be an inevitable phenomenon as the degradation is not likely to occur uniformly over the lug of the deformed bar.

The effect of cyclic loading on the pullout behavior of the rebar is shown in Figure 5. The cyclic loading reduced the maximum bond stress and corresponding slip by 19% and 8%, respectively, compared to those of the specimen subjected to a simple monotonic loading. It is noted that two curves become essentially identical beyond a slip value of 7 mm, corresponding to the frictional resistance branch. The area under the bond stress–slip curve up to the maximum bond stress was 41% smaller in the cyclic loading case than that of the monotonic case. Meanwhile, the area under the entire curve was 14% smaller in the cyclic loading case than that of the monotonic case, indicating that the low-level cyclic loading affects the pre-peak bond–slip behavior more than the post-peak. 

## 3. Experimental Data-Driven Model

The experimental data obtained in this study suggest no model currently available in the literature would not properly capture the bond–slip behavior of a rebar in concrete subjected to low-level cyclic loading with an existing damage; existing numerical bond–slip models consider strain softening due to large crack formation around rebars. Additionally, initial stiffness degradation due to the pre-existing damage on bond capacity must be addressed in the numerical model. In this section, a new numerical model is suggested for low-level cyclic bond–slip behavior.

### 3.1. Simplification of Low-Level Cyclic Bond-Slip Behavior

**Figure 8:**
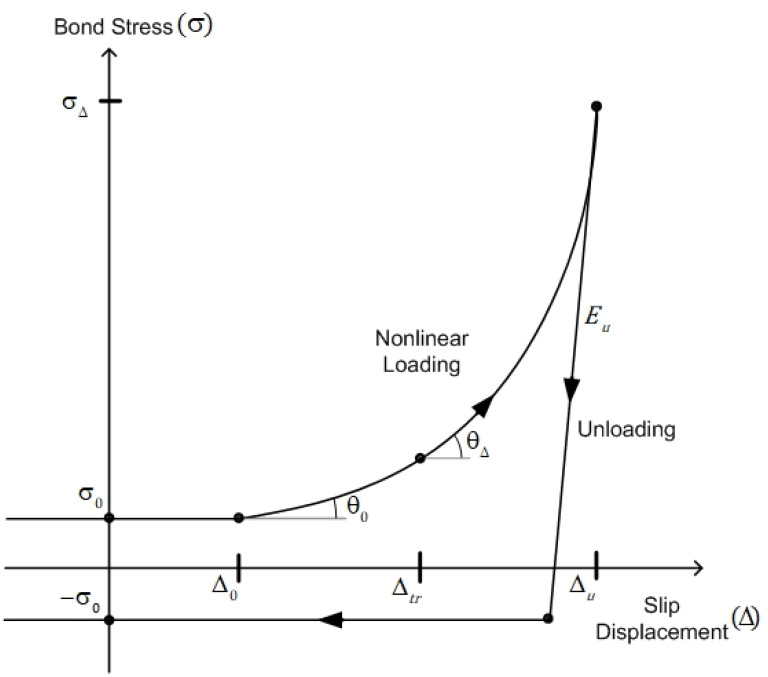
Cyclic nonlinear loading and unloading model.

**Figure 9:**
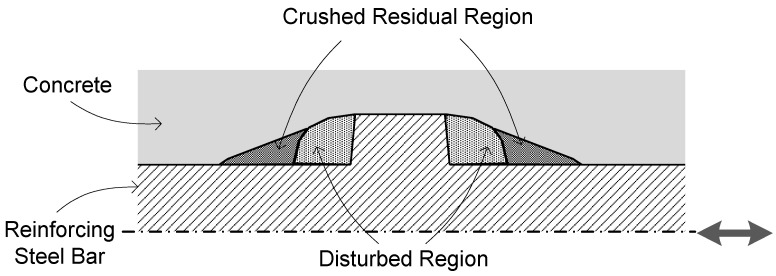
Disturbed and crushed residual regions surrounding steel bars during cyclic loading.

The experimental cyclic curves are simplified as in Figure 8 for the suggested model, where the nonlinear loading curve is characterized by two control points ( $\Delta_{0}$ , $\theta_{0}$ ) and ( $\Delta_{tr}$ , $\theta_{\Delta }$ ). The first control point, $\Delta_{0},$ represents the damaged bond range limit in which a reinforcing steel rebar slides with the minimum bond stress, $\sigma_{0}$ , and is evolved with loading cycles. From the experimental results, it can be reasonably assumed that disturbed and residual regions near the rib of a steel bar exist during each cycle stage as depicted in Figure 9. It is the disturbed region where steel bars slip backward and forward in the damaged region with the minimum bond stress, while the nonlinear loading behavior is due to the onset of the residual region. The peak stress, $\sigma_{\Delta },$ at each cycle is also evolved and gradually decreased to represent the degradation on bond strength and stiffness. The bond stress is unloaded as the elastic unloads with the stiffness of $E_{u}$ . It is noted that the experimental results show the unloading stiffnesses for the pulling direction, which is the initial loading direction, and the opposite (pushing) direction are different. The unloaded bond stress is bounded by the minimum bond stress, $\sigma_{0}$ , in the opposite direction and keeps constant until the slip displacement reaches $\Delta_{0}$ in the opposite direction to represent the slip with the minimum bond stress in the disturbed region.

### 3.2. Formulation of Bond–Slip Model for Unloading/Reloading

The loading curve is suggested in the form of a second-order polynomial to represent both the pre-damaged initial and subsequential cyclic bond–slip behavior:

(1) $$\sigma =\mathrm{sgn}\left(\Delta \right)\left(b_{2}\Delta^{2}+b_{1}\Delta -b_{0}\right)$$

where three coefficients are determined from the slopes at two control points ( $\Delta_{0}$ , $\theta_{0}$ ) and ( $\Delta_{tr}$ , $\theta_{\Delta }$ ) in Figure 8. The coefficients are determined for the initial cycle stage as:

$$b_{0}=0$$

$$b_{1}=\theta_{0}$$

$$b_{2}=\left(\theta_{\Delta }-\theta_{0}\right)/\left(2\Delta_{tr}\right)$$

where $\Delta_{0}=0$ , and two stiffnesses $\theta_{0}$ and $\theta_{\Delta }$ are determined from the experimental data. During the initial cycle, stiffness degradation appears on the loading curve after the stiffness reaches $\theta_{\Delta }$ . In the present study, such degradation is modeled as the hypoelastic stress formulation and Equation (1) is replaced if $d\sigma /d\Delta >\theta_{\Delta }$ :

(2) $$\dot{\sigma }=(1-\frac{\left|\Delta \right|}{\Delta_{lim}})\dot{\Delta }$$

where $\Delta_{lim}$ is the slip displacement limit for the present model, after which the bond strength drops rapidly and the low-level cyclic loading assumption is not valid.

After the first cycle the coefficients are differently set to consider the slip with the minimum bond stress in the disturbed region and degradation on strength:

$$b_{0}=\sigma_{0}$$

$$b_{1}=\theta_{0}$$

(3) $$b_{2}=\left\{\sigma_{\Delta }-\sigma_{0}-\theta_{0}\left(\Delta_{u}-\Delta_{0}\right)\right\}/{\left(\Delta_{u}-\Delta_{0}\right)}^{2}$$

where the parameter, $\Delta_{0}$ , and the peak bond stress $\sigma_{\Delta }$ are evolved with loading cycles and extracted from experimental results.

In the present study, the growth–saturation formulation is suggested to be used for the evolution of the damaged bond range limit parameter:

$$\Delta_{0}=\gamma \Delta_{\max}$$

$$=\underset{N}{\gamma \max}(\Delta_{u})$$

(4) $$\gamma =\gamma_{0}+\frac{\alpha_{1}{\left(N-1\right)}^{2}}{\beta_{1}+{\left(N-1\right)}^{2}}$$

where *N* is the cycle number, which is counted if the slip displacement exceeds $\Delta_{0}$ in each direction. The three parameters, $\gamma_{0},$ $\alpha_{1}$ and $\beta_{1}$ in Equation (4) can be calibrated from the experimental data. A similar form of the growth–saturation formulation is also used to represent the monotonically decreasing evolution of the peak stress due to the cyclic degradation of the bond strength:

$$\sigma_{\Delta }=\omega \sigma_{\max}$$

(5) $$\omega =1-\frac{\alpha_{2}{\left(N-1\right)}^{2}}{\beta_{2}+{\left(N-1\right)}^{2}}$$

where $\sigma_{\max}$ is the maximum bond stress in the previous loading cycles and *N* is set to one whenever the maximum is newly updated. The parameters, $\alpha_{2}$ and $\beta_{2}$ in Equation (5) are also calibrated from the experimental data.

For unloading, an elastoplastic-type incremental bilinear model is used:

(6) $$\dot{\sigma }=E_{u}\dot{\Delta }$$

where $\dot{\sigma }$ is the bond stress increment and $\dot{\Delta }$ is the slip displacement increment. The unloading stiffness can be defined asymmetrically for each slip direction from the observation of the experimental results:

$$E_{u}=\left\{\begin{array}{c} E_{+}\\ 0\\ E_{-} \end{array}\right.\;\;\;\;\;\;\;\;\;\;\;\begin{array}{c} \Delta \geq 0\;and\;\left(\sigma +\dot{\sigma }\right)\geq -\sigma_{0}\\ otherwise\\ \Delta \leq 0\;and\;\left(\sigma +\dot{\sigma }\right)\leq +\sigma_{0} \end{array}$$

### 3.3. Numerical Simulation and Model Validation

**Figure 10:**
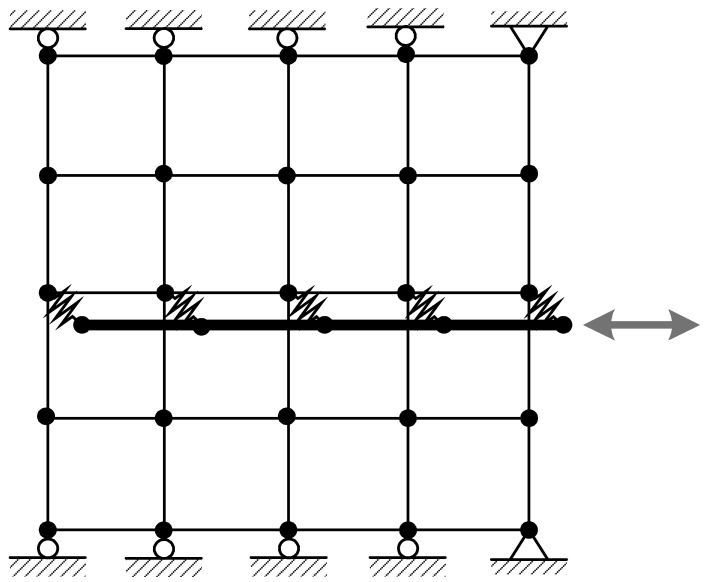
Finite element mesh for reinforced concrete specimen and boundary conditions.

The parameters in the suggested numerical model are determined from the experimental results in Section 2. To validate the numerical model, the experiment is numerically simulated. The finite element mesh and boundary conditions are depicted in Figure 10. Plane stress elements are used for ambient concrete and truss elements are used for steel reinforcing bars. For modeling presently developed bond–slip behavior, zero-length link elements are used. Since the concrete material and nonlinear contact behavior in bond–slip regions under a small amount of cyclic slip displacements is readily considered in the numerical model, the concrete is modeled as an elastic one. The slip displacement limit parameter in Equation (2) is $\Delta_{lim}=0.6$ mm. From the experimental data analysis, $\gamma_{0}=0.3$ , $\alpha_{1}=0.6,$ and $\beta_{1}=2.0$ in Equation (4), and $\alpha_{2}=0.6$ and $\beta_{2}=1.85$ in Equation (5) are used. The cyclic displacement range is the same as in the experiment: $\Delta_{u}$ = 0.5 mm. For the stiffness parameters in Equation (6), $\theta_{\Delta }=18.85$ MPa/mm, $\theta_{0}=0$ , $E_{+}=94.25$ MPa/mm, and $E_{-}=37.70$ MPa/mm, are set based on the experimental results.

**Figure 11:**
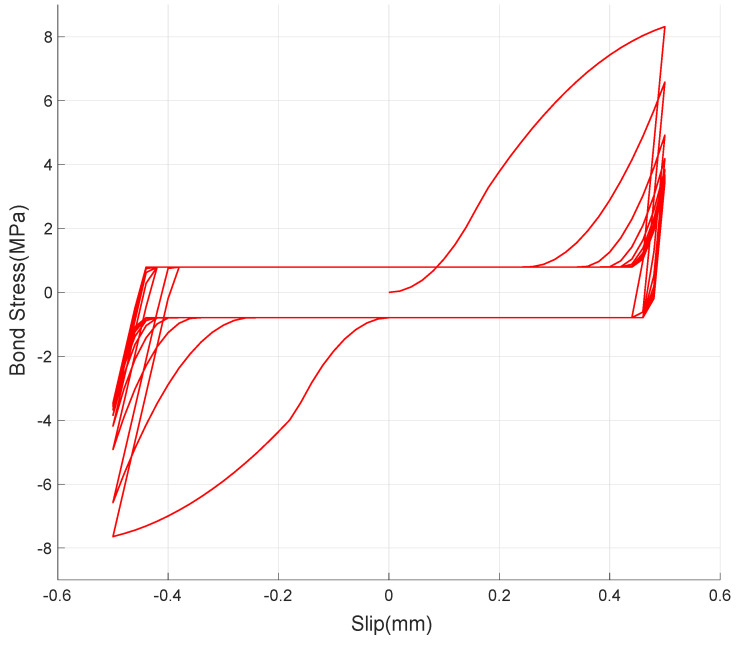
Numerically simulated cyclic behavior of bond stress.

**Figure 12:**
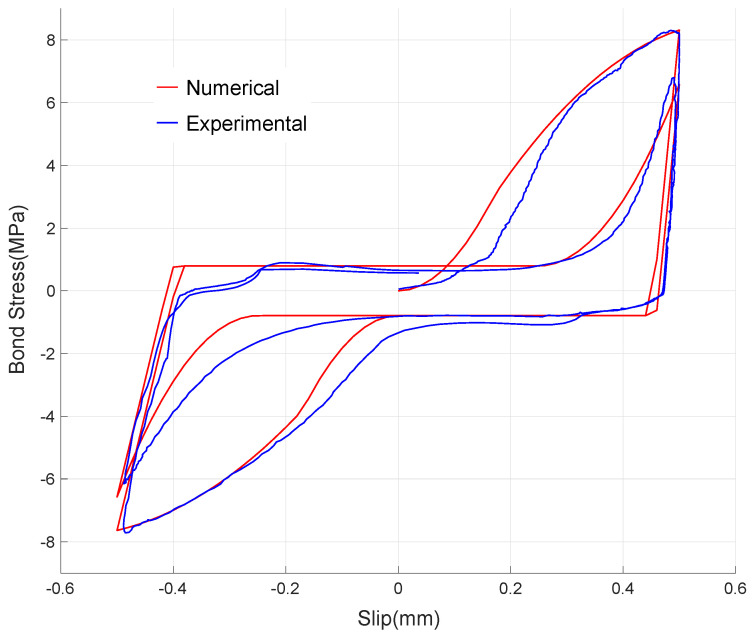
Numerical bond–slip result comparing with experimental one for two cycles.

Full 10 cycles as in the experiment are simulated and the numerical result is plotted in Figure 11. It can be observed that the result appropriately simulates the experimental bond–slip behavior discussed in Section 2. To evaluate the reproduction capability of the presently suggested numerical model, the first two cycles are closely compared in Figure 12. It is shown that the numerical model realistically represents the pre-damaged bond behavior by providing the initial damage on stiffness. It is also verified that the evolution of the damaged bond–slip boundary, $\Delta_{0}$ , and the peak bond stress, $\sigma_{\Delta }$ , during the second loading path is appropriately reproduced. 

To check the validation of the numerical model, relative errors between the experimental, and numerical results on the peak bond stresses in the pulling direction are evaluated and listed in Table 3. The maximum relative error is 7.98% at the sixth cycle and the average relative error for all the 10 cycles is 3.21%, showing that the suggested numerical bond–slip model simulates the experiment within the acceptable accuracy. The overall results show that the presently suggested numerical bond-slip model realistically reproduces the experimental results and can be used in simulating low-level cyclic bond–slip behavior with the pre-existing damage on bond capacity due to cyclic service loading.

## 4. Discussion

Based on the current study, it was found that cyclic loads, which are well below the elastic limit, can cause a significant change in the initial part of the bond stress–slip curve. Such initially degraded bond behavior is especially important in low-to-moderate intensity earthquake analysis and reflected in the proposed numerical model, which is suited for the analysis of old reinforced concrete structures. The following conclusions can be derived:

(1) The low-level cyclic load, (±)0.3 × S_m_ can cause significant degradation to steel rebar-concrete bond in the first three cycles, but the rate of degradation diminishes rapidly in subsequent cycles.
(2) Damage caused by cyclic loading affects the pullout behavior of the steel rebar. Measurable difference can be seen in the maximum bond stress, corresponding slip, and slope of descending branch. But the effect of the cyclic loading on the frictional resistance was negligible.
(3) A new numerical model has been developed to simulate the effect of low-level cyclic load on the bond–slip behavior. The results obtained from the simulation showed good agreement with that from the experiment.

## 5. Implications and Limitations

Bond behavior of steel reinforcing bar in concrete has been studied by several researchers; however, to the best of the authors’ knowledge, no attempts have been made to consider the effect of pre-existing damage in the bond. The implications and limitations of this study can be drawn as follows:

(1) Since the accumulated damage to the bond between steel rebar and concrete, due to low-level cyclic loading, such as the moving service loads or small earthquakes, can compromise the seismic performance of reinforced concrete structures, its effect on bond behavior of steel bar in concrete need to be understood. In the present experimental study this pre-existing damage effect was verified, and it was discovered that the initial loading behavior is significantly different from the conventional ones.
(2) The developed numerical bond–slip model can be embedded into a nonlinear finite element analysis procedure to simulate the realistic effect of low-level cyclic loading with the pre-existing bond stiffness degradation. This can contribute to enhancing the accuracy of performance evaluation of existing deteriorated reinforced concrete structures subjected to low-to-moderate intensity earthquakes.
(3) In order to refine the proposed numerical model, further experimental studies are needed to investigate the effects of other parameters, such as rebar size and concrete strength, pertaining to hysteresis effects. Additionally, the proposed numerical model is not applicable to predict structural behavior beyond low-level cyclic loads, as the slip range considered in the model is limited up to the peak bond strength. Consequently, the softening of bond behavior cannot be represented, and should be addressed in the future development of a high-level cycle model.

## Figures and Tables

**Table 1:** Chemical composition of steel bar (% mass).

Element	C	Si	Mn	P	S	Cu	N	C_eq_
% mass	0.27	0.13	0.49	0.014	0013	0.33	0.01	0.40

**Table 2:** Concrete mix proportions.

Air Content	W/C	Water	Cement	Fine Aggregate	Coarse Aggregate
4.5%	48.2%	111 kg	213 kg	902 kg	902 kg

**Table 3:** Relative errors between the experimental and numerical results on peak stresses.

Cycle	Experimental (MPa)	Numerical (MPa)	Error (%)
1	8.29	8.31	0.28
2	6.75	6.58	2.57
3	4.89	4.92	0.58
4	4.24	4.18	1.32
5	4.17	3.85	7.76
6	3.99	3.67	7.98
7	3.83	3.57	6.77
8	3.55	3.51	1.19
9	3.52	3.47	1.53
10	3.51	3.44	2.07

## Data Availability

Data sharing is not applicable to this article.

## Nomenclature

$S_{m}$	average local slip corresponding to the maximum bond stress in the experiment
τ	bond stress in the experiment
Δ	slip displacement in the numerical model
$\Delta_{0}$	damaged bond range limit in slip displacement
$\Delta_{u}$	maximum slip displacement at the current cycle
$\Delta_{tr}$	calibration control point in slip displacement
$\Delta_{lim}$	slip displacement limit
$\Delta_{\max}$	maximum value of $\Delta_{u}$ at the past and current cycles
$\theta_{0}$	bond-slip stiffness at $\Delta_{0}$
$\theta_{\Delta }$	bond-slip stiffness at $\Delta_{tr}$
σ	bond stress in the numerical model
$\sigma_{\max}$	maximum bond stress in the previous loading cycle
$\sigma_{0}$	minimum bond stress after damage
$\sigma_{\Delta }$	peak bond stress
γ	ratio of $\Delta_{0}$ to $\Delta_{\max}$
$\gamma_{0}$	initial value of γ
$\alpha_{1}$	first parameter for the γ formulation
$\beta_{1}$	second parameter for the γ formulation
$\alpha_{2}$	first parameter for the ω formulation
$\beta_{2}$	second parameter for the ω formulation
ω	ratio of $\sigma_{\Delta }$ to $\sigma_{\max}$
$E_{u}$	unloading stiffness
N	cycle number
